# Tyramine-Functionalized Alginate-Collagen Hybrid Hydrogel Inks for 3D-Bioprinting

**DOI:** 10.3390/polym14153173

**Published:** 2022-08-03

**Authors:** Sung Dong Kim, Subin Jin, Sumin Kim, Donghee Son, Mikyung Shin

**Affiliations:** 1 Department of Biomedical Engineering, Sungkyunkwan University (SKKU), Suwon 16419, Korea; sdkim3451@gmail.com; 2 Center for Neuroscience Imaging Research, Institute for Basic Science (IBS), Suwon 16419, Korea; subinjin@g.skku.edu; 3 Department of Intelligent Precision Healthcare Convergence, Sungkyunkwan University (SKKU), Suwon 16419, Korea; ally0618@naver.com; 4 Department of Electrical and Computer Engineering, Sungkyunkwan University (SKKU), Suwon 16419, Korea; 5 Department of Superintelligence Engineering, Sungkyunkwan University (SKKU), Suwon 16419, Korea

**Keywords:** 3D-bioprinting, tyramine-conjugated alginate, collagen, hybrid hydrogel bioink

## Abstract

Extrusion-based 3D-bioprinting using hydrogels has exhibited potential in precision medicine; however, researchers are beset with several challenges. A major challenge of this technique is the production of constructs with sufficient height and fidelity to support cellular behavior in vivo. In this study, we present the 3D-bioprinting of cylindrical constructs with tunable gelation kinetics by controlling the covalent crosslinking density and gelation time of a tyramine-functionalized alginate hydrogel (ALG-TYR) via enzymatic reaction by horseradish peroxidase (HRP) and hydrogen peroxide (H_2_O_2_). The extruded filament was crosslinked for a second time on a support bath containing H_2_O_2_ to increase fidelity after printing. The resulting tubular construct, with a height of 6 mm and a wall thickness of 2 mm, retained its mechanical properties and had a maximum 2-fold swelling after 2 d. Furthermore, collagen (COL) was introduced into the ALG-TYR hydrogel network to increase the mechanical modulus and cell cytocompatibility, as the encapsulated fibroblast cells exhibited a higher cell viability in the ALG-TYR/COL construct (92.13 ± 0.70%) than in ALG-TYR alone (68.18 ± 3.73%). In summary, a vascular ECM-mimicking scaffold was 3D-bioprinted with the ALG-TYR/COL hybrid hydrogel, and this scaffold can support tissue growth for clinical translation in regenerative and personalized medicine.

## 1. Introduction

Extrusion-based 3D-bioprinting is a novel method for fabricating artificial tissues and bioengineered scaffolds for tissue regeneration (e.g., blood vessels, cardiac muscles, and dental pulp) [1,2,3]. Biomedical hydrogels are utilized as “bioinks,” which are 3D-printable cell encapsulated crosslinked polymers that retain large quantities of water [4]. Such hydrogel inks consist of either synthetic or natural polymers with versatile chemical moieties to form a water-swollen crosslinked network. Bioinks from natural biomaterials are frequently preferred over synthetic ones because of their inherent biomimetic extracellular matrix (ECM) structures [5,6,7]. ECM-mimicking approaches incorporate specific ratios and physical spacings of glycosaminoglycans and fibrous proteins (e.g., elastin/collagen) that constitute the native ECM of cardiovascular tissues [8]. In particular, alginate hydrogel ink, due to its non-immunogenicity, low cost, and ease of gelation, has been extensively studied for ECM-mimicking tissue-engineered solutions such as bioprinting strategies [9,10,11]. However, because of the relatively narrow range of the rheological properties of alginate gels, researchers have often encountered difficulties in optimizing their mechanical properties for bioprinting [12]. In addition, the bioinert property and poor cell adhesion of alginate cause a lack of differentiation and the proliferation of angiogenic cells [13].

To precisely control the mechanical properties and functionalize the bioinert surface of the alginate hydrogel, its chains are often modified to react to specific crosslinkable moieties (e.g., azide/alkyne groups for click reactions, photo-crosslinkable methacrylate, and polyphenols) [14,15,16,17,18,19]. Among them, the photo-crosslinking strategy has been extensively studied due to their excellent mechanical properties and good shape fidelity. However, free radicals generated by photo-triggered cleavage of the chemical can cause high cytotoxicity [20]. Therefore, while mechanical strength is vital to bioprinting success, cell-friendly, noncytotoxic crosslinking strategies are optimal for soft tissue bioprinting such as ECM-mimetic scaffolds. In our previous report, we investigated whether polyphenol modification such as tyramine conjugation improved the adhesion and mechanical properties of hydrogels [21]. As a result, the structural integrity of the fabricated 3D-scaffold is enhanced as the tyramine concentration is proportional to the gel crosslinking rate and storage modulus, and inversely related to the degradation rate [22,23]. In addition, these phenol-conjugated alginate backbones can be oxidized to further improve the mechanical stability of the structure via covalent crosslinking between horseradish peroxidase (HRP) and hydrogen peroxide (H_2_O_2_) [21]. Nonetheless, tyramine conjugation does not contribute to the formation of a bioactive, ECM-mimetic environment (i.e., cell proliferation and differentiation) [23]. Therefore, collagen, which is ubiquitous in the body and essential for the ECM, is often incorporated into functionalized hydrogels [24].

Obtaining bioactivity along the surface is vital for creating functional hydrogels with an ECM-mimicking environment. Thus, in addition to the biochemical function of the hydrogel surface induced by phenols, collagen-induced bioactivity can have an important role in enhancing cellular behavior [25,26]. Because of its excellent biocompatibility with native tissues and amine and carboxyl functional groups that form crosslinks, collagen is widely used for tissue regeneration [24]. Furthermore, as a thermosensitive polymer, collagen polymerizes at body temperature. As a result, collagen fiber assembly can improve the mechanical and nutritional transport properties of hydrogels in vivo [26].

**Figure 1:**
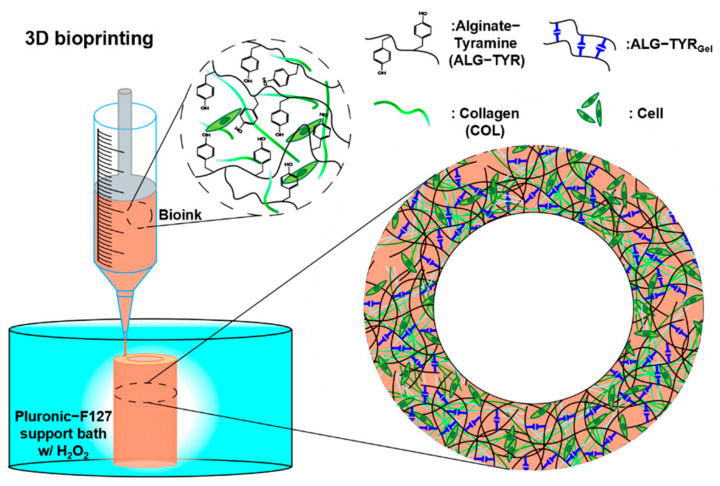
A schematic of the 3D-bioprinting process. The bioink formulation contained alginate-tyramine polymer chains and collagen, which that can be crosslinked to form a mechanically stable, high-resolution structure with homogeneously distributed cells.

The incorporation of multiple materials is common in bioprinting. Therefore, the hybrid network of functionalized alginate and collagen are expected to exhibit a synergistic effect as a bioink [27]. To date, many bioinks are available that can be 3D-printed in meshes or struts. However, when increasing the height to the relevant tissue dimensions, the shape fidelity of the injected hydrogel cannot bear the weight of the subsequent layers, making 3D-bioprinting difficult for clinical transition. In this study, we synthesized and characterized a covalently crosslinked tyramine-conjugated alginate bioink functionalized with collagen (Figure 1); our bioink exhibited biophysical properties due to covalently crosslinked phenols and biochemical properties due to collagen. The 3D-bioprinted structure had high shape fidelity in dimension printing (~6 mm height), and the scaffold’s cell adhesion sites enabled the proliferation of cells.

## 2. Materials and Methods

Sodium alginate (ALG, medium viscosity), tyramine hydrochloride (TYR), N-hydroxysuccinimide (NHS), MES solution, sodium hydroxide (NaOH), HRP, and hydrogen peroxide solution (H_2_O_2_) were purchased from Sigma-Aldrich (St. Louis, MO, USA). N-(3-dimethylaminopropyl)-N’-ethylcarbodiimide hydrochloride (EDC) was purchased from Tokyo Chemical Industry (Tokyo, Japan). Sodium Chloride (NaCl) was purchased from Daejung (Siheung, Korea). Collagen type I from rat tail (COL) was purchased from Corning Inc. (Corning, NY, USA). Dulbecco’s modified Eagle’s medium (DMEM), penicillin-streptomycin, and fetal bovine serum (FBS) were purchased from Gibco (Waltham, MA, USA).

### 2.1. Synthesis and Characterization of Tyramine-Functionalized Alginate Hydrogel (ALG-TYR)

The amine group of the tyramine moiety was conjugated to the carboxylic group of the alginate backbone using EDC/NHS coupling chemistry. First, 1 g of ALG was dissolved at a final concentration of 1% *w*/*v* in MES buffer solution (0.1M) and adjusted to pH 4.7 through a drop-wise addition of NaOH. Subsequently, 5 mmol each of EDC, NHS, and TYR (at a molar ratio of 1:1:1) was dissolved in the buffer solution and mixed overnight at room temperature in a nitrogen gas environment. Once homogeneously mixed and conjugated, the solution was dialyzed with a 6–8 kDa MWCO membrane in 100 mM NaCl for 2 d and deionized distilled water for 4 h to remove any unreacted molecules from the mixture. After dialysis, the tyramine-conjugated alginate (ALG-TYR) was obtained through lyophilization. 

The degree of tyramine substitution (DOS) of ALG-TYR was first analyzed using Varian Oxford 300 MHz ^1^H NMR spectroscopy (Varian, Palo Alto, CA, USA). The polymer (1% *w*/*v*) was dissolved in deuterium oxide (D_2_O). The integral value of the peak (6.7–7.1 ppm) regarding hydrogen atoms present in the aromatic ring of TYR was compared with those in the ALG backbone (4.8–5.3 ppm) [28,29]. Second, the DOS was further analyzed using Agilent 8453 UV–Vis spectroscopy (Agilent Technologies, Santa Clara, CA, USA). A calibration curve of TYR was initially obtained as y = 0.0081x−0.0003 (x = TYR concentration, y = absorbance at the wavelength of 274 nm). Then, the absorbance (A_274_) of the ALG-TYR solutions at different concentrations was evaluated in reference to the TYR linear equation to evaluate the DOS.

### 2.2. Rheological Measurements

The rheological characterization of each hydrogel sample was performed using a Discovery Hybrid Rheometer (TA Instruments, New Castle, DE, USA). The machine parameters were kept constant with a 20 mm parallel plate geometry and a testing gap of 500 µm. The storage modulus (G’), loss modulus (G”), and damping factor (tan δ) of the hydrogels were determined using a frequency sweep test at 1% strain from 0.01 to 100 Hz. A frequency-time test at 1 Hz was performed to determine the gelation time of the hydrogel from covalent crosslinking. An oscillation test was performed in the shear rate range of 0.01 to 100 s^−1^ to determine the shear viscosity of the hydrogels. 

#### 2.2.1. Crosslinking of ALG-TYR Hydrogel

The ALG-TYR hydrogel was covalently crosslinked using HRP and H_2_O_2_ enzymatic reactions. First, different H_2_O_2_:TYR molar ratios (0.01, 0.05, 0.1, and 0.2) were prepared to determine the crosslinking density by observing the changes in G’ and tan δ. Different concentrations of HRP (0.1, 0.5, and 1 U/mL) were prepared after characterizing the H_2_O_2_:TYR ratio to determine the gelation time of the hydrogel in G’ and G” vs. time. Finally, different concentrations of the ALG-TYR solution (1.5 to 3% *w*/*v*) with an equivalent molar ratio of H_2_O_2_:TYR and a concentration of HRP were tested to determine the changes in the crosslinking density and viscosity, which translated directly to the printability of the ink. 

#### 2.2.2. Functionalization of Bioactivity with Inclusion of Collagen

Collagen solutions of 1, 2, and 3 mg/mL were first polymerized at pH 7.4 and 37 °C, as described in the manufacturer’s protocol. A rheological study was performed to determine a suitable concentration for the bioactivity functionalization of the ink. The selected concentration was premixed with the ALG-TYR hydrogel prior to the HRP-induced crosslinking. The resulting hybrid hydrogel was used as the final ink solution before cell encapsulation.

### 2.3. Swelling Studies of Printed Construct

The newly printed construct was first weighed for the initial mass (*W_I_*). Subsequently, the construct was soaked in 1x phosphate buffer saline (PBS), and the mass of each sample at the time intervals (30 min to 2 d) was weighed for swelling mass (*W_S_*). The swelling ratio was calculated using the following equation:

(1) $$\mathrm{Swelling}\;\mathrm{ratio}=\frac{W_{S}-W_{I}}{W_{I}}\times 100\%$$

### 2.4. 3D-Bioprinting Tubular Scaffold

#### 2.4.1. Construct Design

A cylinder with a height of 6 mm and diameter of 10 mm was designed using a blender and printed using the NewCreatorK 3D-printing software (Rokit Healthcare, Seoul, Korea). The temperature of the injecting solution was maintained at 5 °C and printed on a 37 °C bed with a 20% (*w*/*v*) Pluronic F-127 support bath containing H_2_O_2_ for secondary crosslinking. The temperatures of the solution and support bath were maintained throughout the fabrication process. 

The following printing parameters were optimized and fixed: 26 G needle, 1 mm/s printing speed, and 0.3 mm layer-to-layer distance. 

#### 2.4.2. Cell Encapsulation and Bioprinting

Before cell encapsulation, all materials were sterilized using germicidal UV light with the wavelength ranging from 100 to 280 nm (λ_max_ = 253.7 nm) for 30 min. For the cell culture, the mouse fibroblast cells (L929) were maintained under standard cell culture conditions (37 °C, 5% CO_2_ cell culture incubator) in DMEM supplemented with 10% FBS and 1% penicillin-streptomycin. Cultured cells were washed with DPBS and dissociated with 0.05% trypsin-EDTA. The dissociated cells were resuspended in the prepared 5 °C un-crosslinked ink containing optimized concentrations of ALG-TYR, COL, and HRP at a cell density of 1 × 10^6^ cells/mL and gently mixed. Immediately after cell encapsulation, H_2_O_2_ was added and mixed to induce primary crosslinking. Subsequently, the partially crosslinked bioink was 3D-printed under the same conditions as those mentioned in Section 2.4.1.

### 2.5. Cell Viability and Histological Analysis

A live/dead assay was used to measure the viability of the L929 mouse fibroblast cells encapsulated in the printed scaffolds 1, 3, and 7 d after 3D printing. Fluorescent images were obtained from Leica DMi 8 fluorescent microscope (Leica, Wetzlar, Germany). In the 3D printed constructs, 2 µM calcein AM and 4 µM ethidium homodimer-1 were stained at room temperature for 30 min. The stained constructs were moved to a 20 Ø confocal dish and evaluated using a Leica TCS SP8 STED confocal microscope (Leica, Wetzlar, Germany). The live/dead cells on each mentioned date were counted and analyzed using ImageJ to evaluate the cell viability and proliferation.

### 2.6. Statistical Analysis

All results are presented as the mean ± standard deviation. All statistical analyses were performed using the analysis of variance using GraphPad Prism 7. Significant differences were defined as ns for ‘not significant’, * *p* < 0.05, ** *p* < 0.01, *** *p* < 0.001, and **** *p* < 0.0001. At least three independent trials were performed, unless stated otherwise.

## 3. Results and Discussion

### 3.1. Characterization of ALG-TYR Hydrogels

**Figure 2:**
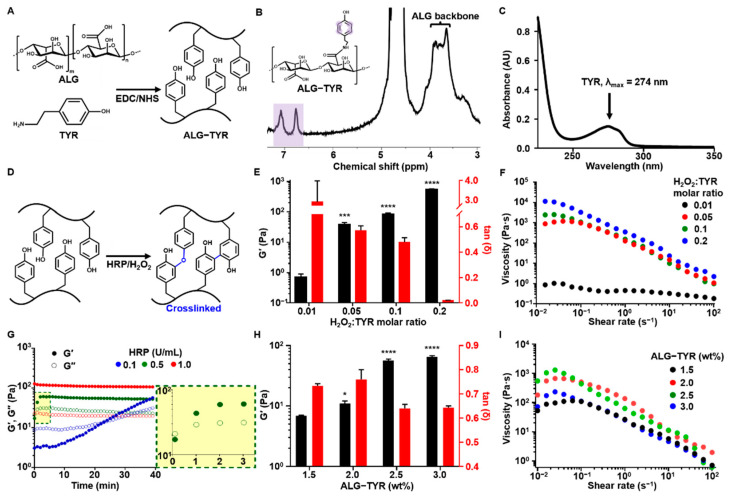
The ALG-TYR hydrogel characterization. (**A**) The ALG-TYR conjugation mechanism with the EDC/NHS coupling reaction. The carboxyl group in the alginate binds to the amine group of the tyramine to form a phenol-functionalized backbone chain. (**B**) The ^1^H NMR analysis of ALG-TYR. Different marked peaks (purple) represent hydrogen atoms in tyramine. (**C**) UV–Vis spectroscopy at the 274 nm peak (tyramine). (**D**) The enzymatic crosslinking mechanism of ALG-TYR chains induced by HRP/H_2_O_2_. (**E**) The storage modulus and tan δ of different H_2_O_2_:TYR ratios at 1% strain and 1 Hz. (**F**) The changes in viscosity depend on the applied shear rate on different H_2_O_2_:TYR ratios. The significance of each G’ data was compared with that of the H_2_O_2_:TYR ratio at 0.01. (**G**) The gelation time kinetics observed through changes in the HRP concentrations. Highlighted region: 0.5 U/mL HRP. (**H**) The storage modulus and tan δ of different concentrations of ALG-TYR at 1% strain and 1 Hz. The significance of each G’ data was compared with that of 1.5% ALG-TYR. (**I**) Changes in viscosity depend on the applied shear rate on different concentrations of ALG-TYR. (*n* = 3, mean ± SD) (* *p* < 0.05, *** *p* < 0.001, **** *p* < 0.0001).

To manufacture a hydrogel scaffold that functions as an ECM-mimicking scaffold for tissue regeneration, we fabricated an injectable hybrid hydrogel comprising interpenetrating polymers. First, the TYR-conjugated ALG was prepared through the modification of alginate with tyramine via an EDC/NHS coupling reaction and lyophilization (Figure 2A). The solid form of ALG-TYR was dissolved in PBS. The DOS of tyramine was first analyzed using ^1^H NMR spectroscopy by comparing the integral value of the protons in the aromatic region (6.5–7 ppm) to that of the protons in the polysaccharide (3–4.5 ppm). The resulting DOS was calculated to be 5.8% (Figure 2B). Additionally, the DOS of tyramine was analyzed using UV–Vis spectroscopy by comparing the absorbance values of ALG-TYR to pristine tyramine at 274 nm. UV–Vis spectroscopy yielded a DOS of 3.8% (Figure 2C). The difference in DOS obtained from ^1^H NMR (~5.8%) and UV–Vis (~3.8%) spectroscopies might be due to the overestimation issue of the proton signals at ^1^H NMR [30].

For further discussion, in the crosslinking reaction, an increase in TYR conjugation may result in an increase in crosslinking density and zeta potential. ALG is an anionic polysaccharide due to the presence of negatively charged carboxyl groups. The TYR is tethered onto the carboxyl groups, decreasing the number of negatively charged COO^−^, which has a role of electrostatic repulsion among ALG polymeric chains. An excessively high DOS can result in the folding/aggregation of polymeric chains by an increase in the net charge and highly dense crosslinking by phenol groups, and easily fragmented gelation not for injectable/printable gelation [31]. Therefore, we synthesized our ALG-TYR to an optimal DOS of approximately 5% to achieve an injectable hydrogel with a tunable crosslinking density.

#### 3.1.1. Effect of H_2_O_2_ Concentrations on Printability

The phenols of ALG-TYR are covalently crosslinked through the enzymatic reaction of HRP in the presence of H_2_O_2_, forming their dimers with either C–C or C–O linkages (Figure 2D) [32,33]. Such two different types of dimers can be randomly generated, however, the C–C linked ones are dominant. The injectability and mechanical strength of the crosslinked hydrogel were primarily determined by optimizing the H_2_O_2_ ratio (0.01 to 0.2) to the phenol moieties of the conjugated tyramine [21]. The viscoelastic properties of each sample were characterized at a constant concentration of 2.5% (*w*/*v*) ALG-TYR and 0.5 U/mL for HRP (Figure 2E). As expected, G’ exhibited an increasing trend as the concentration ratio of H_2_O_2_ increased. Values of 0.71 ± 0.19, 38.10 ± 6.45, 83.30 ± 8.62, and 556 ± 0.45 ± 20.41 Pa were observed for a H_2_O_2_:TYR ratio of 0.01 (0.043 mM of H_2_O_2_), 0.05 (0.216 mM of H_2_O_2_), 0.1 (0.432 mM of H_2_O_2_), and 0.2 (0.864 mM of H_2_O_2_). No significant increase in the modulus of the hydrogel was observed when the ratio was further increased from 0.2 (data not shown). That is, their crosslinking degree might be almost ~90–100% for the hydrogel prepared at a molar ratio of H_2_O_2_ to a TYR of 0.2 or above. This was because most of the phenols in the hydrogel had already been crosslinked; thus, there was an excess of unreacted H_2_O_2_ [34]. Therefore, approximately 500 Pa was the maximum achievable mechanical strength of our injectable hydrogel at 2.5% (*w*/*v*), which could be achieved at a H_2_O_2_:TYR ratio between 0.1 and 0.2. 

Another factor to consider when optimizing H_2_O_2_:TYR is tan δ. A low tan δ indicates stiffer gel formation regardless of the magnitude of G’ or G”. As the concentration of H_2_O_2_ increased, the tan δ decreased, due to the higher crosslinking density. With higher stiffness, the hydrogel has lower permeability and higher crosslinking degree, thereby reducing cell proliferation and viability in the bioink. Generally, a tan δ value below 0.2 exhibits granular and brittle textures when extruded, thereby limiting cell movements in the construct [35]. Therefore, a tan δ value of at least 0.2 is required for bioinks, which is any formulation with a H_2_O_2_:TYR ratio of 0 to 0.1. In our experiment, a H_2_O_2_:TYR ratio of 0.1 satisfied this requirement at a tan δ value of 0.48 ± 0.029.

The flow behavior analysis exhibited shear thinning behavior in all of the hydrogel samples, which corresponded to the injectability of the hydrogel (Figure 2F) [36]. Shear thinning behavior demonstrates the dissociation of reversible dynamic linkages present in the polymer network [37]. Of the samples, a H_2_O_2_:TYR ratio of 0.05 to 0.2 exhibited a viscosity of approximately 10^1^ to 10^4^ Pa·s across all shear rates. Generally, for 3D extrusion printing, ink viscosity is in the range of 10^1^ to 10^5^ Pa·s [38]. Except for the sample with a H_2_O_2_:TYR ratio of 0.01, all of the samples were within this range; thus, any parameters between the H_2_O_2_:TYR ratios of 0.05 and 0.2 were deemed printable with a continuous filament. To induce sufficient crosslinking density, cell viability, and printability, we selected a H_2_O_2_:TYR ratio of 0.1 as the final crosslinking ratio used for the pre-crosslinking bioink. 

#### 3.1.2. Effect of HRP Concentrations on Printability

In the translation of a hydrogel to gel ink for printing, the gelation time must be optimized for sufficient crosslinking time of the scaffold after printing to control the precision of the printing process. The gelation time of HRP-mediated crosslinking (e.g., the cross-point of G’ and G” values) was measured at enzyme concentrations of 0.1, 0.5, and 1 U/mL. The concentrations of the ALG-TYR and H_2_O_2_ were maintained at 2.5% (*w*/*v*) and at a H_2_O_2_:TYR ratio of 0.1 (0.43 mM), respectively. Rheological studies indicated that 1 U/mL was the fastest in gelation time as the hydrogel crosslinked immediately after the addition of HRP (Figure 2G). However, 0.5 U/mL of HRP exhibited a slower gelation time within 1 min (Figure 2G, yellow box) and 0.1 U/mL exhibited the slowest gelation time at 25 min. The incubation time of the polymer/HRP/H_2_O_2_ mixture temporarily affects their G’ value while gelation takes place. Unless the G’ value reaches the plateau level (e.g., complete gelation), the mechanical modulus of the mixture gradually improves as the incubation time of the mixture (e.g., reaction time) increases [39]. Because most of the printings for this experiment were conducted within 3 min at most, the HRP of 0.5 U/mL was selected for ease of handling and an appropriate crosslinking time after extrusion. 

#### 3.1.3. Effect of ALG-TYR% Concentrations on Printability

The viscoelasticity of the hydrogel depends on the DOS as well as the polymer concentrations to be crosslinked. Significant crosslinking variability can occur even with the same H_2_O_2_:TYR ratio at different ALG-TYR concentrations [40]. In our study, 2.5% and 3% ALG-TYR exhibited an improved modulus compared with 1.5% and 2.0% (Figure 2H). In addition, the overall viscosity of the hydrogel increased with an increase in the ALG-TYR content within the printable range (Figure 2I). Therefore, we concluded that an ALG-TYR concentration of at least 2.5% is required for a printable ink based on the increase in elastic modulus and viscosity of the network.

### 3.2. Characterization ALG-TYR/COL Hybrid Hydrogels

**Figure 3:**
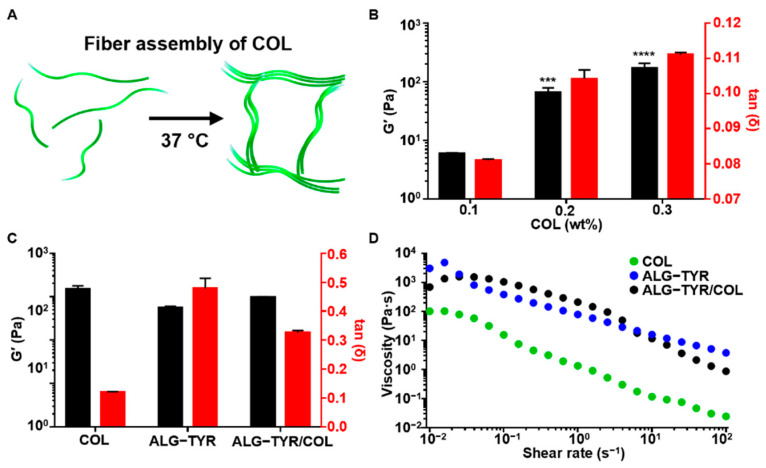
The characterization of the ALG-TYR/COL hybrid hydrogel. (**A**) An illustration of collagen polymerization at 37 °C. Unpolymerized collagen molecules floated freely until the temperature was increased and the collagen fibers assembled. (**B**) Storage modulus and tan δ of the collagen substrates at 1% strain and 1 Hz. The significance of each G’ data was compared with that of the 0.1% collagen. (**C**) The comparison of the storage modulus and tan δ across COL, ALG-TYR, and ALG-TYR/COL at 1% strain and 1 Hz. Significance of each G’ data was compared with that of COL. (**D**) Changes in the viscosity depending on the applied shear rate on COL, ALG-TYR, and ALG-TYR/COL. (*n* = 3, mean ± SD) (*** *p* < 0.001, **** *p* < 0.0001).

To prepare the ALG-TYR/COL hydrogel for stable adhesion and cell growth of the completed hydrogel scaffold, collagen, which is present in the ECM, was polymerized with the ALG-TYR hydrogel. It is well-known that collagen polymerizes into a fibrous structure as the temperature is increased to 37 °C at a neutral pH of 7.4 (Figure 3A). First, different concentrations of pristine collagen at 1, 2, and 3 mg/mL were neutralized to pH 7.4 through the addition of NaOH and studied for their gelation kinetics. The collagen fibers were uniformly polymerized at approximately 20 min to G’ values of 5.89 ± 0.26, 65.69 ± 12.89, and 168.29 ± 37.76, respectively (Figure 3B). In addition, the main role of collagen in hybrid polymer networks of ALG-TYR and collagen was to introduce bioactivity to the ALG-TYR rather than participate in the viscoelastic properties of the gel. Interestingly, however, the introduction of collagen above 0.2% (2 mg/mL) caused it to have higher mechanical properties (G’) but a significantly lower tan δ than those of the crosslinked ALG-TYR. Accordingly, ALG-TYR and COL may have a synergistic crosslinking density to maintain the mechanical modulus for high fidelity while obtaining optimal tan δ for cell compatibility. The synergistic stiffness of the hybrid hydrogel was observed by mixing both polymers. Crosslinked ALG-TYR exhibited an increase in storage modulus by 39 ± 7.13 Pa and a decrease in tan δ by 0.15 ± 0.03 with the introduction of COL (Figure 3C). This indicates that the incorporation of collagen into the network increased the overall stiffness. However, no significant change in viscosity was observed with the addition of COL (Figure 3D). This indicates that the viscosity of the network was mostly governed by the ALG-TYR chains rather than by the COL fibers. 

### 3.3. 3D-Printability the ALG-TYR/COL Hydrogel Ink

**Figure 4:**
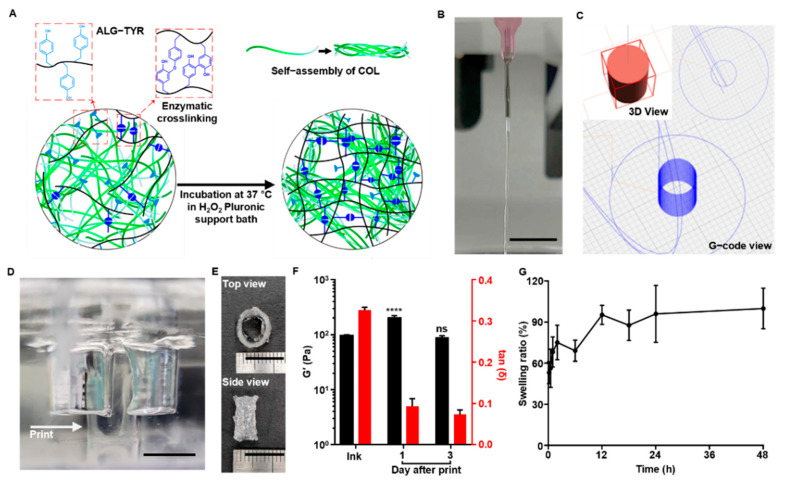
The 3D-printing ALG-TYR/COL hybrid ink. (**A**) An illustration of how the ink formulation is partially crosslinked between phenols and undergo additional crosslinking after printing. The additional crosslinking is induced by the increased temperature and higher concentration of H_2_O_2_ in the printing bed. (**B**) Continuous filament in 26G needle extrusion was an indication of appropriate injectable hydrogel for 3D-bioprinting. (**C**) Design of the tubular construct. A 10 mm diameter and a height of 6 mm with a 2 mm wall thickness was designed to mimic human artery dimensions. (**D**) Printing was conducted in a Pluronic F-127 support bath. The printed construct (indicated with arrow) was crosslinked by H_2_O_2_ inside the bath to increase the crosslinking density of the final construct. (**E**) The construct was printed with a high resolution at the designed dimensions. (**F**) Storage modulus and tan δ of the ink before printing and the printed construct over 1 and 3 d at 1% strain and 1 Hz. Significance of each G’ data was compared with that of the ink before printing. (**G**) Swelling test of the constructs from 10 min to 2 d. (Scale bar = 10 mm) (*n* = 3, mean ± SD) (**** *p* < 0.0001, ns = not significant).

The synergistic stiffness of the ALG-TYR/COL hybrid hydrogel enabled the ink network to obtain a controlled crosslinking density for injectability and fidelity. The pre-crosslinking of ALG-TYR using HRP/H_2_O_2_ in the preparation of the ink enabled appropriate injectability as partial phenol moieties were oxidized (Figure 4A). Upon extrusion on a 37 °C Pluronic F127 support bath containing H_2_O_2_:TYR at a ratio of 0.1 (0.43 mM of H_2_O_2_), collagen molecules underwent fiber assembly due to the increased temperature, and ALG-TYR stiffened due to more crosslinking. 

As the printing was conducted, the optimized ink formulation had a stable filament at the extrusion site. Optimal printability can be defined as a continuous filament without fluctuation during injection [41]. We extruded the crosslinked hydrogels with a 26 G needle to evaluate the printability (Figure 4B) and the continuous filament was observed, further enabling the minimization of the mechanical stress to cells to be encapsulated. 

To evaluate the printability of the ALG-TYR/COL ink into a 3D structure with high fidelity and high cell compatibility, we designed a tubular structure with a 6 mm height and 10 mm diameter with a wall thickness of 0.6 mm (Figure 4C) and printed in the prepared support bath (Figure 4D). To prevent the collagen from fiber assembly before injection, the needle temperature was maintained at 5 °C. When printed on the 37 °C Pluronic F-127 bed containing 0.43 mM of H_2_O_2_, the construct was left to crosslink inside an incubator for 1 h before collection to completely crosslink both ALG-TYR and COL. As a result, the initially printable hydrogel ink with a H_2_O_2_:TYR ratio of 0.1 further crosslinked under the support bath with an additional H_2_O_2_:TYR ratio of 0.1 (e.g., a total H_2_O_2_:TYR ratio of 0.2). This strategy resulted in the optimal printing of the construct shape as designed (Figure 4E). In addition, the mechanical modulus (e.g., G’ value) of the printed construct increased up to 198.24 ± 19.75 Pa (tan δ at 0.093 ± 0.015) after a 1-d incubation in PBS from the initial ink value of 95.13 ± 3.50 Pa (tan δ at 0.325 ± 0.007) (Figure 4F). This indicated that the H_2_O_2_ inside the support bath was successfully reacted with residual phenol groups and bolstered the fidelity of the construct. After 3-d of incubation, the G’ value of the construct was 85.96 ± 7.91 Pa (tan δ at 0.072 ± 0.010), which was lower than that at 1 d, but the original shape was retained. Such a decrease in the mechanical modulus might be due to swelling of the hydrogel, which can further affect cellular spreading and proliferation in the construct [42]. 

The swelling properties of the construct are important when describing the final printing resolution. The ease of diffusion of the nutrients and molecules is essential in 3D-hydrogel structures for cell survival and tissue regeneration [43]. However, an excessively high swelling can disrupt the spatial arrangement of polymer chains due to the penetration of liquid between the crosslinked networks [44]. Therefore, the balance between water uptake and the ability to retain water content without deformation of the structure can be studied through a swelling test. In our swelling test, our ALG-TYR/COL hydrogel experienced an initial increase of 53.10 ± 7.97% in weight within 10 min and a maximum increase of 99.86 ± 14.87% after 2 d of soaking in PBS (Figure 4G). At any time point, none of the samples were dissolved, and the weight did not change significantly after 2 d of swelling, indicating that the scaffold could absorb water up to twice its initial weight while retaining its architecture.

### 3.4. Cytocompatibility and Cell Proliferation of ALG-TYR/COL Scaffold

**Figure 5:**
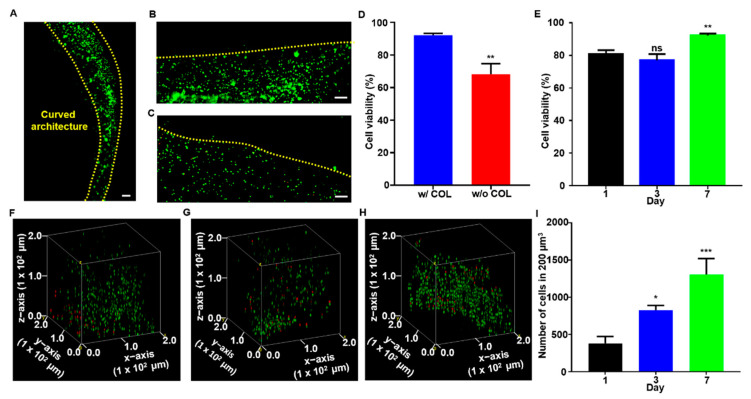
The cell cytocompatibility and histological analysis. (**A**) Seeded cells proliferated along the curved structure of the construct. Live/dead staining of (**B**) ALG-TYR/COL construct and (**C**) ALG-TYR construct without COL. Red arrows indicate dead cells (red) stained with EthD-1 (scale bar = 100 µm). (**D**) The cell viability of constructs with and without COL were measured. (**E**) The cell viability of ALG-TYR/COL constructs over 1, 3, 7 d were analyzed. Significance was compared with cell viability on d 1. (**F**–**H**) Confocal images stacked into volume at 200 µm^3^ on d 1, 3, and 7 using the Volume Viewer plugin in ImageJ. (**I**) Number of cells in 200 µm^3^ of d 1, 3, and 7 for the proliferation of cells. Significance was compared with the number of cells on d 1. (Scale bars = 100 µm) (*n* = 3, mean ± SD) (* *p* < 0.05, ** *p* < 0.01, *** *p* < 0.001, ns = not significant).

To demonstrate the feasibility of the ALG-TYR/COL hydrogel ink for 3D-bioprinting, mouse fibroblast (L929) cells were encapsulated in the polymer solution, and cell viability and proliferation within the printed construct were investigated as a function time (Figure 5). In general, high shear stress can be applied to the cells during extrusion of the bioink, frequently leading to disruptions of the cellular membrane and low cell viability after printing. That is, to enhance cell viability during printing, the injectability of the bioink is very important. Thus, we chose an ink formulation with a H_2_O_2_:TYR ratio of 0.1 (G’ = 83.30 ± 8.62 Pa, tan δ = 0.48 ± 0.029) for great injectability. Meanwhile, alginate is known to have bioinert properties that prevent cell adhesion [45], and alginate chains should be functionalized to improve the cell-matrix interactions [46]. Nonetheless, various surfaces such as protein-based binding sites are required in addition to the phenol moieties to further enhance multiple cell-binding sites on the scaffold surface [47]. In our bioink, we introduced collagen as the protein responsible for increasing cell adhesion. We fabricated 3D-constructs with and without COL for cell viability to test how collagen could increase the bioactivity of our scaffold (Figure 5A–E).

After 7 d of incubation, the scaffolds were stained with calcein AM (green) to indicate live cells and EthD-1 (red) to indicate dead cells. All samples exhibited a high cell viability in general and homogenous proliferation along the printed construct in a curved line (edges marked by yellow dashed line) as designed (Figure 5A). Through the evaluation using a fluorescent microscope, only a small signal of red at the edges of the scaffold (yellow dashed lines) was observed when collagen was included in the network (Figure 5B) whereas a high content of dead cells was clearly visible in the construct printed without collagen (Figure 5C). Overall, the cell viability was decreased from 92.13 ± 0.70% to 68.18 ± 3.73% when collagen was excluded, which indicated that constructs functionalized with collagen are essential for cell survivability (Figure 5D). Furthermore, with the collagen included, the cell viability was 80.57 ± 1.53, 76.93 ± 2.21, and 92.13 ± 0.70% on d 1, 3, and 7, respectively (Figure 5E). Throughout the culture period, we observed that the majority of cells survived the printing and culturing processes. While the difference in cell viability between d 1 and 3 was not statistically significant, it was lower than that on d 7, indicating the gradual increase in the rate of proliferation after printing. For the first three days, the trypsinized cells experienced high stress from printing and adhered to the hydrogel surface, resulting in slow proliferation rate and necrosis [48]. When the cells were stabilized in the construct, a few necrotic cells were present, resulting in a 11.56 ± 2.27% difference in cell viability between d 1 and 7. Finally, cells were counted in a fixed volume (200 µm^3^) of the construct to quantitatively measure cell proliferation over 7 d of culturing (Figure 5F–H). Cell counts at 380 ± 53, 825 ± 37, and 1307 ± 213 were observed on d 1, 3, and 7, respectively. The proliferation results indicated that an average of 140 cells proliferated per day within the specified volume, and that there was more than a 3-fold increase over 7 d of culture. The result might be because a slight decrease in the mechanical modulus of the swollen construct (Figure 4F) helps cellular spreading and proliferation within the polymeric network.

In conclusion, our experiments showed that the conjugation of tyramine to alginate to functionalize the polymer to prepare injectable hydrogels with cell adhesion sites was bolstered by the addition of collagen, which clearly increased the fidelity of our construct and overall cell viability. Therefore, the construct was printed in dimensions that mimicked a human vessel, 10 mm in diameter, 6 mm in height, and 2 mm in wall thickness and remained in shape after several days of culturing. Compared with conventional bioinks for extrusion-based 3D printing, our system has several advantages. First, we were able to control viscoelasticity at multiple stages of the process: (i) DOS of ALG-TYR can be mediated by the conjugation process due to the EDC/NHS coupling reaction; (ii) enzymatic crosslinking via HRP/H_2_O_2_ offers versatility when preparing injectable hydrogels; and (iii) chain molecular density via different ALG-TYR and COL concentrations finely tune the crosslinking density and viscosity. Our bioink is a simple print-and-culture formulation that does not require additional photo-crosslinking or surface treatment that could potentially damage cells. Finally, bioactivity achieved from tyramine and collagen offers high cell viability and proliferation that can be used to produce a wide range of 3D-printable tissue engineering applications. Therefore, both the mechanical and biochemical properties required for 3D bioprinting were achieved using our simple yet flexible ALG-TYR/COL hybrid hydrogel.

## 4. Conclusions

In this study, we fabricated an injectable hydrogel comprising tyramine-conjugated alginate and collagen. The crosslinking strategies for this hybrid hydrogel include enzymatic crosslinking using HRP and H_2_O_2_ to oxidize the phenols and thermo-responsive crosslinking of collagen at body temperature. The viscoelastic properties, or the injectability, of the ALG-TYR/COL matrix were dominated by enzymatic crosslinking between the phenol moieties in tyramine, while the collagen fibril network within the matrix assists in mechanical strength and cell attachment. Therefore, the crosslinking parameters for the ALG-TYR hydrogel were first characterized (at 2.5% (*w*/*v*) of the polymer, 0.5 U/mL of HRP, and 0.43 mM of H_2_O_2_) and COL (at 0.2% (*w*/*v*)) was incorporated into the ink subsequently. The L929 cells were encapsulated inside the hydrogel and printed immediately on a Pluronic F-127 support bath containing 0.43 mM of H_2_O_2_ to induce secondary crosslinking for shape fidelity. The printing was conducted on a 6 mm high tubular construct that mimicked the ECM of a human blood vessel. Subsequent rheology studies and histology analyses of the construct indicated that our hydrogel is printable, retains high fidelity after printing, and has up to 92.13 ± 0.70% cell survivability that can be upscaled and translated for further study in vascular tissue engineering applications.

## Data Availability

Data is contained within the article.
